# Spinal Interneurons With Dual Axon Projections to Knee-Extensor and Hip-Extensor Motor Pools

**DOI:** 10.3389/fncir.2020.00007

**Published:** 2020-03-12

**Authors:** Khuong H. Nguyen, Thomas E. Scheurich, Tingting Gu, Ari Berkowitz

**Affiliations:** ^1^Department of Biology, University of Oklahoma, Norman, OK, United States; ^2^Cellular and Behavioral Neurobiology Graduate Program, University of Oklahoma, Norman, OK, United States

**Keywords:** synergy, propriospinal, knee extensor, hip extensor, retrograde labeling

## Abstract

The central nervous system (CNS) may simplify control of limb movements by activating certain combinations of muscles together, i.e., muscle synergies. Little is known, however, about the spinal cord interneurons that activate muscle synergies by exciting sets of motoneurons for different muscles. The turtle spinal cord, even without brain inputs and movement-related sensory feedback, can generate the patterns of motoneuron activity underlying forward swimming, three forms of scratching, and limb withdrawal. Spinal interneurons activated during scratching are typically activated during all three forms of scratching, to different degrees, even though each form of scratching has its own knee-hip synergy. Such spinal interneurons are also typically activated rhythmically during scratching motor patterns, with hip-related timing. We proposed a hypothesis that such interneurons that are most active during rostral scratch stimulation project their axons to both knee-extensor and hip-*flexor* motoneurons, thus generating the rostral scratch knee-hip synergy, while those interneurons most active during pocket scratch stimulation project their axons to both knee-extensor and hip-*extensor* motoneurons, thus generating the pocket scratch knee-hip synergy. The activity of the entire population would then generate the appropriate synergy, depending on the location of sensory stimulation. Mathematical modeling has demonstrated that this hypothesis is feasible. Here, we provide one test of this hypothesis by injecting two fluorescent retrograde tracers into the regions of knee-extensor motoneurons (more rostrally) and hip-extensor motoneurons (more caudally). We found that there were double-labeled interneurons, which projected their axons to both locations. The dual-projecting interneurons were widely distributed rostrocaudally, dorsoventrally, and mediolaterally within the hindlimb enlargement and pre-enlargement spinal segments examined. The existence of such dual-projecting interneurons is consistent with the hypothesis that they contribute to generating the knee-hip synergy for pocket scratching. The dual-projecting interneurons, however, were only about 1% of the total interneurons projecting to each location, which suggests that they might be one of several contributors to the appropriate knee-hip synergy. Indirect projections to both motor pools and/or knee extensor-dedicated interneurons might also contribute. There is evidence for dual-projecting spinal interneurons in frogs and mice as well, suggesting that they may contribute to limb motor control in a variety of vertebrates.

## Introduction

The central nervous system (CNS) may simplify limb movement coordination through use of muscle synergies, i.e., simultaneous activation of certain combinations of motoneurons and thereby muscles (Ting and Mckay, 2007; Bizzi et al., 2008; Tresch and Jarc, 2009; Giszter, 2015; Bruton and O’Dwyer, 2018; Del Vecchio et al., 2019). In principle, the use of muscle synergies could greatly reduce the number of parameters that the CNS must control. Little is known, however, about which CNS neurons generate muscle synergies or how (Levine et al., 2014; Giszter, 2015).

The turtle spinal cord can coordinate the same set of limb muscles to generate several kinds of limb movements, even in the absence of brain input and movement-related sensory feedback (Berkowitz, 2010; Stein, 2018). These movements include forward swimming, three forms of scratching, and limb withdrawal. Scratching is a rhythmic movement in which a limb rubs repeatedly against a site on the body surface. Each form of scratching targets a different region of the body surface. Knee extension generates the force to rub the limb against the body. The timing of knee extension within the forward (flexion) and backward (extension) hip movement cycle differs for each form of scratching, causing the limb to rub against a distinct region of the body in each case (Mortin et al., 1985). In rostral scratching, knee extension occurs during hip *flexion*, while in pocket scratching, knee extension occurs during hip *extension*. Similarly, in immobilized animals, a knee extensor motor nerve burst occurs during each hip *flexor* motor nerve burst in rostral scratching but during each hip *extensor* motor nerve burst in pocket scratching (Robertson et al., 1985). Thus, rostral and pocket scratching have distinct knee-hip synergies.

**Figure 1:**
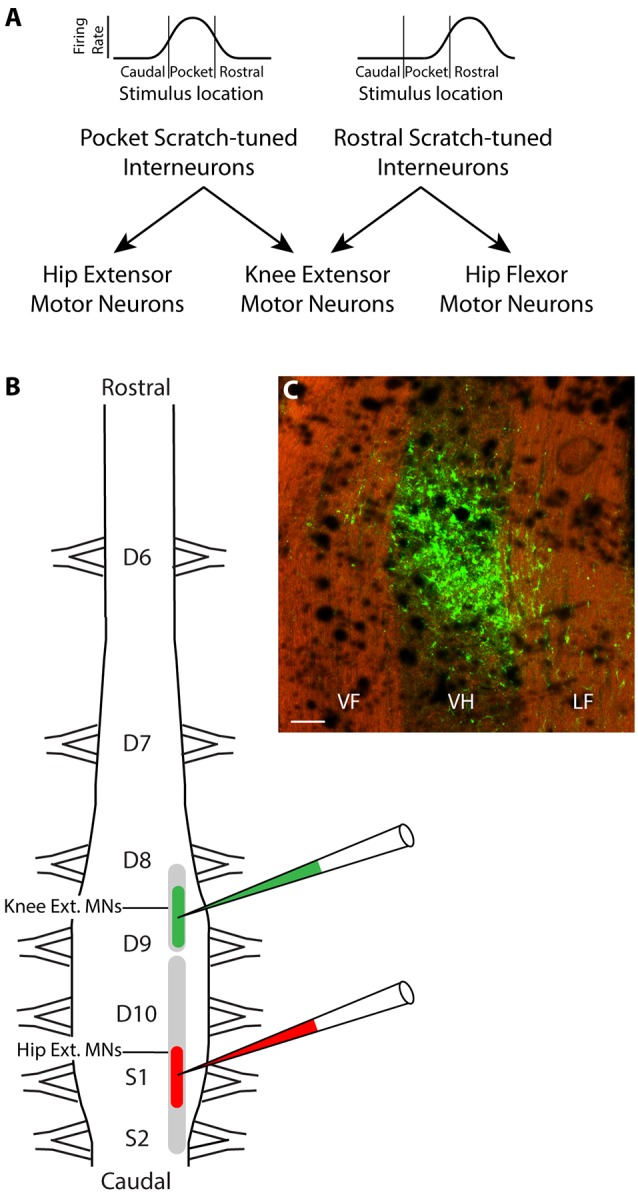
Hypothesis and experimental design. **(A)** Schematic illustration of hypothesized circuit to generate the appropriate knee-hip synergies for rostral and pocket scratching. Rhythmically active spinal interneurons broadly tuned to the rostral scratch receptive field are hypothesized to project axons to both knee-extensor and hip-flexor motoneurons, while those broadly tuned to the pocket scratch receptive field project instead to knee-extensor and hip-extensor motoneurons. **(B)** Experimental design: different fluorescent retrograde tracers were injected into the right ventral horn of rostral D9 (rD9), where knee-extensor motoneuron somata are, and S1, where hip-extensor motoneuron somata are. **(C)** Low-magnification epifluorescence image showing an example of the site of injection of Alexa Fluor 488-dextran amine, in the rD9 right ventral horn, in a horizontal section (rostral is up; lateral is right). Note that the tissue autofluorescence clearly reveals the borders of the gray and white matter. VF, ventral funiculus; VH, ventral horn; LF, lateral funiculus. Scale bar: 100 μm.

Most spinal interneurons activated during one form of scratching are activated during all three forms of scratching, but to different degrees, as each such interneuron is typically broadly tuned to a region of the body surface and the three forms of scratching have different body-surface receptive fields (Berkowitz and Stein, 1994a; Berkowitz, 2001a). Each such interneuron typically is also rhythmically active within a certain phase of the hip activity cycle during all forms of scratching (Berkowitz and Stein, 1994b; Berkowitz, 2001b). We proposed that the appropriate knee-hip synergies for rostral scratching and pocket scratching could be generated by the total population of such interneurons if rostral scratch-tuned interneurons project their axons to both knee-extensor and hip-*flexor* motoneurons, while pocket scratch-tuned interneurons project their axons to both knee-extensor and hip-*extensor* motoneurons (Figure 1A; Berkowitz and Stein, 1994b; Berkowitz, 2001b). The net effect of the population activity would then be expected to bring about the appropriate knee-hip synergy for each location stimulated. For example, tactile stimulation within the receptive field for pocket scratching would activate pocket scratch-tuned interneurons more than rostral scratch-tuned interneurons (or caudal scratch-tuned interneurons), so the net effect of the interneuronal population activity would be to activate the pocket scratch knee-hip synergy, in which knee-extensor motoneurons are active during the hip-extensor motoneuron bursts. Computational simulation has demonstrated the feasibility of this proposed circuit (Snyder and Rubin, 2015).

The turtle spinal cord hindlimb enlargement consists of five segments: dorsal (D) 8–10 and sacral (S) 1–2. The somata of knee-extensor motoneurons and hip-flexor motoneurons largely overlap rostrocaudally, but the somata of knee-extensor and hip-extensor motoneurons do not (Ruigrok and Crowe, 1984). Knee-extensor motoneurons are in the caudal part of the D8 segment and the rostral part of the D9 segment while hip-extensor motoneurons are more caudal, in the caudal D9 through S2 segments. Thus, injections of a retrograde tracer into the ventral horn of the rostral D9 (rD9) segment should label axons projecting to the knee-extensor (but not hip-extensor) motor pool, while injections into the ventral horn of the S1 segment should label axons projecting to the hip-extensor (but not knee-extensor) motor pool. The proposed dual-projecting interneurons that would generate the knee-hip synergy for pocket scratching should project axons to both of these regions.

It is challenging to test this hypothesis experimentally, however. In an initial test, we found that injections of a retrograde tracer into the rD9 ventral horn in one set of animals and the S1 ventral horn in another set of animals gave rise to distributions of labeled spinal interneuron somata that were partly overlapping, especially in the D10 segment (Berkowitz, 2004). This experiment could not demonstrate, however, that individual interneurons project their axons to both motor pools, as these data may have resulted from interneuron somata with a single axon projecting to one motor pool interspersed with interneuron somata with a single axon projecting to the other motor pool.

To test more rigorously the hypothesis that there are dual-projecting interneurons that could generate the knee-hip synergy for pocket scratching, here we injected two different fluorescent dextran amine retrograde tracers (Glover et al., 1986; Novikova et al., 1997; Nissen et al., 2008) into the deep ventral horn of rD9 and S1 in each animal (Figure 1B) and later screened the D6-S2 spinal cord for single-labeled and double-labeled interneurons. Double-labeled interneurons thus had axons projecting to the regions of both the knee-extensor and the hip-extensor motor pools. We found that such dual-projecting interneurons exist, comprising about 1% of the population of interneurons projecting to each region. Dual-projecting interneurons were widely distributed in the spinal cord, rostrocaudally, dorsoventrally, mediolaterally, and both ipsilaterally and contralaterally.

## Materials and Methods

### Surgical and Injection Procedures

Adult red-eared turtles, *Trachemys Scripta elegans* (*n* = 7; 450–750 g; both sexes), were anesthetized and surgically prepared as described previously (Berkowitz, 2004). Briefly, the spinal cord was exposed and transected between the D2 and D3 dorsal roots, then also exposed from the D6 through the S2 segment. The meninges were torn on the dorsal surface of rD9 and S1, where dye injections were later made into the ventral horn. All surgical procedures were approved by the Institutional Animal Care and Use Committee of the University of Oklahoma.

Micropipettes were pulled from 1.0 mm O.D. borosilicate glass containing a filament (Sutter Instrument Company, Novato, CA, USA), using a P-97 puller (Sutter), and backfilled with either 5% Alexa Fluor 488-dextran amine (10,000 MW; Molecular Probes/Invitrogen) or 5% Alexa Fluor 568-dextran amine (10,000 MW; Molecular Probes/Invitrogen) *via* capillary action for ≥30 min. Each micropipette tip was broken to a diameter of 70–150 μm just before use, to counteract clogging. The micropipette was held in a micromanipulator and advanced using a piezoelectric microdrive (EXFO 8200). The micropipette was positioned 70% of the way laterally from the midline to Lissauer’s tract and lowered to a depth of 1,200 μm for the rD9 injection and 1,000 μm for the S1 injection to target motoneurons in the ventral horn (Berkowitz, 2004). Each dye was injected *via* pressure pulses using a Picospritzer III (Parker Hannifin Corporation/General Valve Operation) using 10–30 psi and 10–30 ms per pulse. Pressure pulses were repeated, with 3-min intervals between pulses, until all of the dye was ejected from the micropipette, ~1.0–1.5 μl. After the injection, the micropipette was left in place for 5 min before withdrawing it. Then, the second location was injected using the same procedure but with the other dye.

### Perfusion and Histology

Following a survival period of 21–28 days, during which animals were housed in tubs partly filled with water and tilted to facilitate basking, with 12 h-on/12 h-off incandescent and UV lamps, and fed twice weekly with aquatic turtle food pellets (Zoo Med Laboratories Inc., San Luis Obispo, CA, USA), each animal was deeply anesthetized with pentobarbital (Euthanasia III, Med-Pharmex, Incorporated, 390 mg, i.p.; Berkowitz, 2004). After the animal was non-responsive, the heart was exposed and perfused with 800 ml turtle saline containing 0.1 ml pentobarbital, 0.1% sodium nitrite, and 48 mg heparin, followed by 300 ml cold 4% paraformaldehyde/0.1 M phosphate buffer, pH 7.4 (PB). Then, the D6-S2 spinal cord was removed, pinned in 4% paraformaldehyde/PB for ≥12 h at 4°C, and then transferred to 20% sucrose/PB for ≥6 h at 4°C, and then gelatin-albumin embedding medium for ≥12 h at 4°C. The embedding medium was hardened by the addition of 0.8 ml 50% (w/w) glutaraldehyde. The block was trimmed and the embedded cord frozen-sectioned on a microtome (Microm HM400) at 50 μm horizontally. All sections were kept. The sections were mounted with 2–3 drops of Vectashield with DAPI (Vector Laboratories, Burlingame, CA, USA) and coverslipped, using clear nail polish to seal the edges.

### Identification of Retrogradely Labeled Somata

All sections through the D6-S2 spinal segments were screened for single-labeled and double-labeled interneurons. The sections were photographed and the labeled neurons quantified using a Nikon Optiphot-2 epifluorescence microscope with Alexa Fluor 488 (Chroma Technology IN027764) and mCherry, Texas Red (Chroma IN007115) filter sets and an Olympus DP70 camera. Single-labeled somata did not visibly fluoresce using the other filter set. Somata were counted as retrogradely labeled neurons if they were outside the cluster of labeled somata at the injection site (e.g., Figure 1C), clearly brighter than the background, and had a visible dendrite, axon, or nucleus. Somata were counted as double-labeled if they additionally were brighter than the background using both filter sets. For figures, low-magnification images of selected sections were obtained with a Zeiss Apotome microscope with a 10× 0.3 NA objective. For high-magnification images, selected sections were imaged on a Leica SP8 confocal laser scanning microscope using a 63× 1.40 NA oil objective. An Argon laser with a 488 nm laser line was used to excite the Alexa Fluor 488 with an emission detection window between 500 nm and 550 nm. Similarly, a DPSS 561 nm laser line was used to excite the Alexa Fluor 568 and an emission detection window was set at 590 nm to 640 nm. Z-series of images were acquired *via* sequential scanning. Image contrast was adjusted in Adobe Photoshop for better visualization of cell bodies and fine cellular processes.

## Results

**Figure 2:**
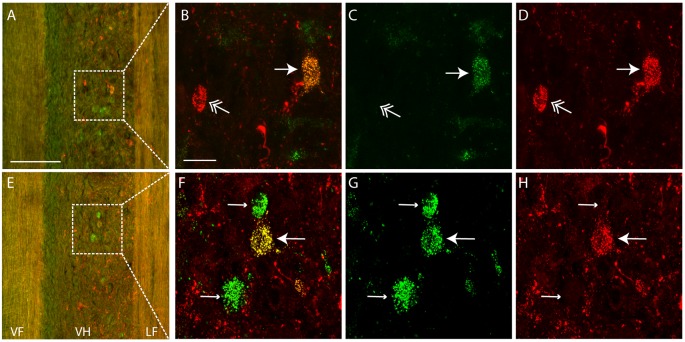
Examples of single- and double-labeled interneurons. **(A,E)** Low-magnification confocal images (at two different focal planes) of a horizontal section (rostral is up, lateral is right) showing single-labeled (green or red) and double-labeled (yellow) interneurons ipsilateral to the injection sites. Note that the tissue autofluorescence clearly reveals the borders of the gray matter. White boxes indicate regions shown at higher magnification in panels **(B–D,F–H)**. **(C,D,G,H)** High-magnification confocal images showing labeling of a subset of interneurons with each dye separately, for the neurons shown in panels **(A,E)**, respectively. **(B,F)** Merged images of panels **(C,D,G,H)**, respectively. Thin arrows indicate interneurons labeled with Alexa Fluor 488-dextran amine only; feathered arrows indicate interneurons labeled with Alexa Fluor 568-dextran amine only; thick arrows indicate double-labeled interneurons. VF, ventral funiculus; VH, ventral horn; LF, lateral funiculus. Scale bars: **(A,E)**, 100 μm; **(B–D,F–H)**, 20 μm.

**Figure 3:**
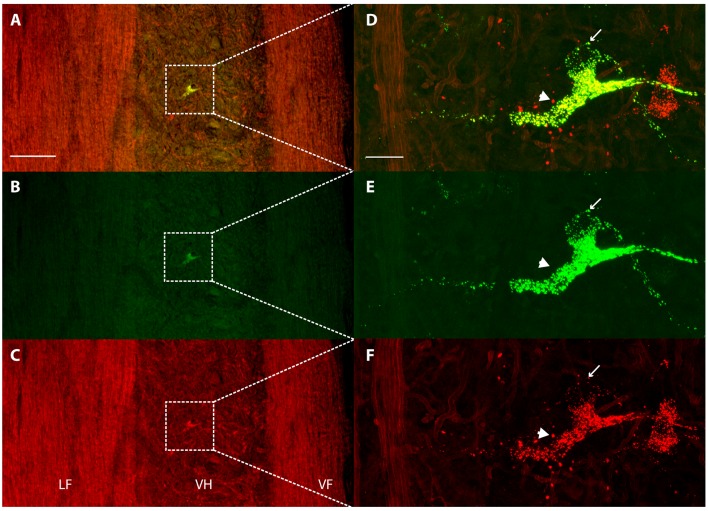
Concentration of retrograde tracers into largely the same cytoplasmic granules. **(A–C)** Low-magnification confocal images of a horizontal section showing labeling of a contralateral ventral horn interneuron with each tracer alone **(B,C)** and the two merged **(A)**. Note that the tissue autofluorescence clearly reveals the borders of the gray matter. White boxes indicate the region shown at higher magnification in panels **(D–F)**. **(D–F)** High-magnification confocal images of the same double-labeled neuron displaying the granular cytoplasmic labeling with each tracer alone **(E,F)** and the two together **(D)**. Note that almost all granules contained both tracers, but some contained only one. The thin arrow indicates a granule that is only labeled with Alexa Fluor 488-dextran amine and the thick arrow indicates a granule that is only labeled with Alexa Fluor 568-dextran amine. Rostral is up; lateral is left. LF, lateral funiculus; VH, ventral horn; VF, ventral funiculus. Scale bars: **(A–C)**, 100 μm; **(D–F)**, 20 μm.

Each dye injection resulted in an approximately spherical cluster of labeled somata typically centered in the lateral part of the ventral horn of rD9 or S1, which effectively marked each injection site (e.g., Figure 1C). Labeled neurons within this cluster at the injection site were not counted as retrogradely labeled, as they may have been labeled *via* their dendrites. Outside this cluster, labeled neurons were found both ipsilaterally and contralaterally, both rostral and caudal to the injection site, as far as 3 cm away. Even within a small region of the gray matter, some neurons could be found that were labeled with one dye only, some with the other dye only, and still others with both dyes (Figure 2). The double-labeled neurons appeared to concentrate the two dyes in largely the same granules within the soma, though a few granules contained just one dye or the other dye (Figure 3).

Both dyes were successfully injected at the intended locations in one preliminary experiment and in six subsequent experiments. In the preliminary experiment, eight double-labeled neurons were found, with one each in the ipsilateral intermediate zones of D7 and D8 and three each in the ipsilateral ventral horns of D9 and D10. The double-labeled neurons comprised 1.2% of the total of neurons retrogradely labeled by the rD9 injection and 4.1% of the total retrogradely labeled by the S1 injection. This preliminary experiment demonstrated that there are spinal neurons with axonal projections to both rD9 and S1.

In the six subsequent experiments, we systematically quantified the numbers and locations of all single-labeled and double-labeled neurons. In three animals, we injected Alexa Fluor 488-dextran amine in rD9 and Alexa Fluor 568-dextran amine in S1, while in the other three animals we injected Alexa Fluor 568-dextran amine in rD9 and Alexa Fluor 488-dextran amine in S1, to avoid any kind of bias in case one dye was more effective than the other. The total number of neurons retrogradely labeled by a single dye ranged from 512 to 7,272, with mean ± SD of 2,039 ± 1,934 and a median of 1,853. The total number of labeled neurons did not depend on either the injection site (rD9: mean, 1,573 ± 1,118; median, 2,008; S1: mean, 2,505 ± 2,542; median, 1,853), or the dye (Alexa Fluor 488: mean, 2,379 ± 2,480; median, 2,008; Alexa Fluor 568: mean, 1,699 ± 1,343; median, 1,853).

The total number of double-labeled neurons in each animal ranged from 3 to 78, with a mean of 19.4 ± 26.5 and a median of 9. The number of double-labeled neurons was higher in animals with more single-labeled neurons, presumably because more dye had been successfully injected in those animals. Thus, we chose to focus on the percentages of retrogradely labeled neurons that were double-labeled, rather than the absolute numbers. In these six experiments, the mean total percentages of double-labeled neurons were 1.18 ± 0.80% of neurons labeled by the rD9 injection (median: 1.1%) and 0.77 ± 0.39% of neurons labeled by the S1 injection (median: 0.66%).

**Figure 4:**
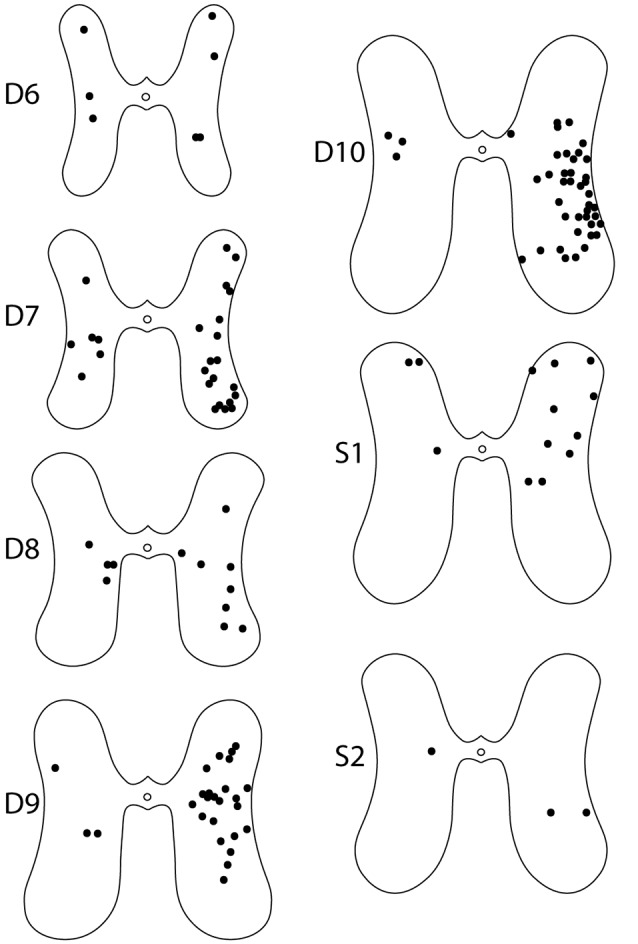
Somata locations of all double-labeled interneurons from six animals charted on schematic cross sections of the hindlimb enlargement (D8–S2) and pre-enlargement (D6–D7) spinal segments. Injections were on the right side in all animals.

**Figure 5:**
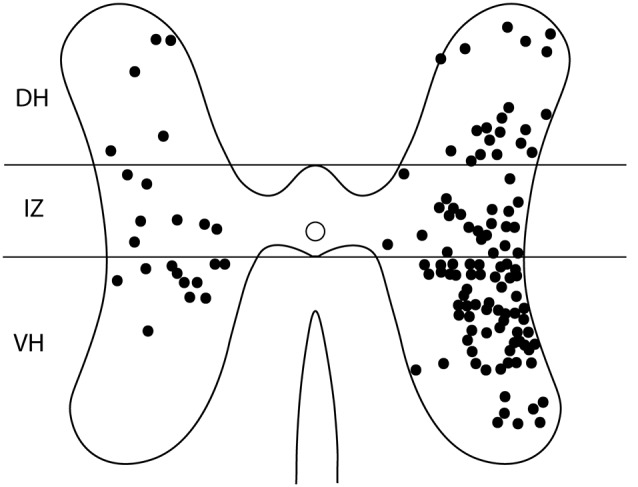
Somata locations of all double-labeled interneurons from six animals charted on a single schematic cross section, to clarify the cross-sectional distribution. DH, dorsal horn; IZ, intermediate zone; VH, ventral horn.

For these six animals, the gray-matter soma locations of all double-labeled neurons were charted on schematic cross-sections for each of the seven spinal cord segments examined: the two pre-enlargement segments, D6 and D7, and the five hindlimb enlargement segments, D8-D10 and S1 (Figure 4). Double-labeled neurons were scattered rostrocaudally, dorsoventrally, and mediolaterally, including all seven segments examined, bilaterally. Figure 5 shows all double-labeled neurons consolidated onto a single schematic cross-section, to better illustrate their dorsoventral and mediolateral locations. Double-labeled neurons were concentrated ipsilaterally, especially in the deep dorsal horn, the intermediate zone, and the dorsal two-thirds of the ventral horn, mostly in the lateral half of the ipsilateral gray matter. Those double-labeled neurons found contralaterally were mostly in the intermediate zone and the dorsal part of the ventral horn.

**Figure 6:**
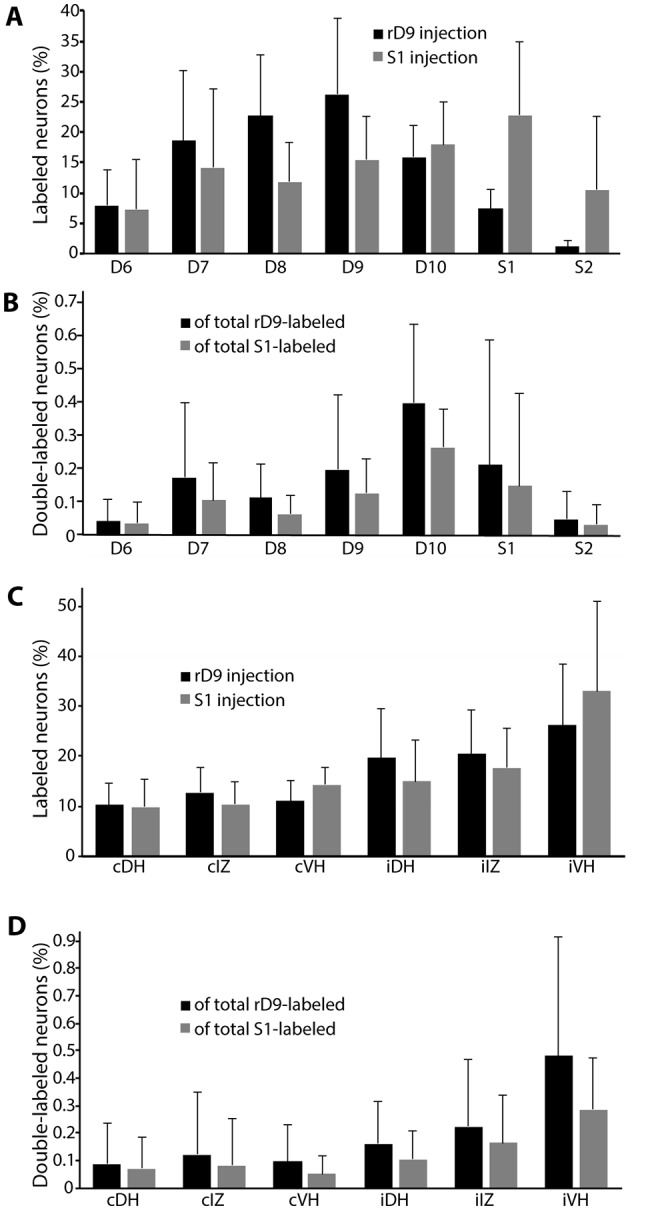
Quantification of distributions of all retrogradely labeled interneurons across six animals. **(A)** Segmental distributions of all interneurons labeled by rD9 (black) and S1 (gray) injections, as percentages of the total number of labeled neurons in each animal. **(B)** Segmental distributions of all double-labeled neurons, as percentages of all retrogradely labeled neurons from the rD9 injection (black) and as percentages of all retrogradely labeled neurons from the S1 injection (gray). **(C)** Cross-sectional distributions of all interneurons labeled by rD9 (black) and S1 (gray) injections, as percentages of the total number of labeled neurons in each animal. **(D)** Cross-sectional distributions of all double-labeled neurons, as percentages of all retrogradely labeled neurons from the rD9 injection (black) and as percentages of all retrogradely labeled neurons from the S1 injection (gray). c, contralateral; i, ipsilateral; DH, dorsal horn; IZ, intermediate zone; VH, ventral horn.

The rostrocaudal distributions of retrogradely labeled neurons appeared to differ for the rD9 and S1 injections (Figure 6A). Neurons projecting to rD9 were concentrated in D7-D10, while the neurons projecting to S1 were more evenly distributed throughout D6–S2. Double-labeled neurons were rostrocaudally concentrated in D7–S1, especially D9–S1, with a plurality in D10 (Figure 6B).

In contrast to the rostrocaudal distributions, the dorsoventral distributions of retrogradely labeled neurons were essentially identical for the rD9 and S1 injections (Figure 6C). Most neurons were found ipsilaterally, especially in the ventral horn, though they were widely distributed in all dorsoventral regions on both sides. Double-labeled neurons were also widely distributed dorsoventrally, both ipsilaterally and contralaterally, though they were concentrated in the ipsilateral ventral horn and, to a lesser extent, the ipsilateral intermediate zone (Figure 6D).

## Discussion

### Characteristics and Distribution of Double-Labeled Interneurons

The total number of retrogradely labeled interneurons in each animal in the current study was on average greater than the number we previously achieved injecting horseradish peroxidase (HRP) into the same locations (Berkowitz, 2004), while the variability in total number of labeled interneurons was similar, which suggests that the current dextran-amine retrograde labeling approach is at least as efficient and reliable as HRP. Also, Nissen et al. (2008) estimated that there are about 7,000 interneurons in a turtle D9 hemisegment that project beyond that segment; we had about one-fourth as many labeled interneurons in each animal in the current study, but of course we were only labeling interneurons that project to the ventral horn, not all projecting interneurons (though we were counting interneuron somata in several segments), so our numbers are approximately what one might expect. Although like many other retrograde tracers, fluorescent dextran amines are taken up by damaged axons in addition to axon terminals, they apparently are not taken up by intact axons of passage (Glover et al., 1986; Novikova et al., 1997). Because we targeted our injections to the gray matter and avoided unnecessary damage, we think that the vast majority of our retrograde labeling was *via* axon terminals.

The neurons in this study that were retrogradely labeled by two dyes injected in two different regions concentrated both dyes in largely the same cytoplasmic granules, though some granules contained just one dye or the other (Figure 3). This suggests that despite accumulating the dyes from different axons, they are usually brought together once in the soma. The fact that some granules contained only one dye, however, also demonstrates that the retrograde movement and soma accumulation of different molecules from different sources are not necessarily linked. In a previous study in which *Xenopus* midbrain motoneurons were retrogradely labeled with two fluorescent dyes coupled to dextran amines applied to the same cut nerve at two different times, the two dyes appeared to be concentrated in the same cytoplasmic granules (Fritzsch and Sonntag, 1991).

The dual-projecting interneuron somata were surprisingly widely distributed rostrocaudally, dorsoventrally, and mediolaterally. This suggests that dual-projecting interneurons might play a variety of roles. The distribution of dual-projecting interneurons was remarkably similar to those found for turtle descending (Berkowitz and Stein, 1994c; Nissen et al., 2008) and ascending (Nissen et al., 2008) propriospinal interneurons generally, which, though widely distributed, were concentrated in the ipsilateral deep dorsal horn, intermediate zone, and dorsal part of the ventral horn, especially laterally, as well as the contralateral intermediate zone and ventral horn, especially medially. This was true despite the fact that many of the interneurons labeled in the current study (those with somata between the two injection sites rostrocaudally) had one ascending axon and one descending axon. Propriospinal interneurons with bifurcating (ascending and descending) axons generally makeup about 6% of the total population of turtle propriospinal interneurons and are found in similar gray-matter locations to those with only an ascending or only a descending axon (Nissen et al., 2008). This similarity of somata locations for propriospinal neurons with single or bifurcating axons has also been found with intracellular recording and dye injection of interneurons activated during scratching (Berkowitz, 2005). The distribution of dual-projecting interneurons ipsilaterally in the current study is also quite similar to the distribution of interneurons activated during scratching and/or swimming (Berkowitz, 2005, 2008), consistent with at least some of the interneurons in the current study being activated during these limb movements, though in the current study we did not test when they were activated. The distribution of double-labeled (as well as single-labeled) interneurons projecting to the ventral horn in this study was also similar to the distributions of interneurons projecting to a single motor pool in adult cats (Grant et al., 1980; Jankowska and Skoog, 1986; Alstermark and Kümmel, 1990; Hoover and Durkovic, 1992), adult rats (Puskár and Antal, 1997; Birinyi et al., 2003), and neonatal mice (Stepien et al., 2010).

### The Possible Role of Dual-Projecting Interneurons in Pocket Scratching

We demonstrate here for the first time that there are individual turtle spinal interneurons that have dual axonal projections to the regions of two hindlimb motor pools: knee extensors and hip extensors, even though the injected regions were about 1 cm apart. Previously, we had demonstrated that a region of the spinal gray matter (especially in the D10 segment) contains interneuron somata that project their axons to each of these regions, though not necessarily to both (Berkowitz, 2004). Our current finding is consistent with the hypothesis that dual-projecting interneurons, strongly activated during pocket scratch sensory stimulation and firing rhythmically with hip-related timing, generate the appropriate knee-hip synergy for pocket scratching, in which each knee-extensor burst occurs during a hip-extensor burst (Berkowitz and Stein, 1994b; Berkowitz, 2001b). Computational simulations have demonstrated that the proposed circuit, with rostral scratch-tuned interneurons projecting to knee-extensor motoneurons and hip-flexor motoneurons and pocket scratch-tuned interneurons projecting to knee-extensor motoneurons and hip-extensor motoneurons, can produce the appropriate knee-hip synergy for each form of scratching, determined by the relative strengths of sensory inputs (Snyder and Rubin, 2015).

There are, however, several caveats to be considered. Neither our current anatomical findings nor the previous mathematical simulations demonstrate that pocket scratching (or rostral scratching) is actually generated by such a circuit. The conclusions of our anatomical experiments are limited by our not knowing: (1) that these interneurons are synaptically connected to the motoneurons we found axonal projections to; and (2) that these interneurons are strongly activated during pocket scratching. Obtaining such data would probably require much more challenging experiments in which interneurons are recorded intracellularly along with two kinds of motoneurons they synapse onto. It is also not clear that the relatively small number of such dual-projecting interneurons we found here (about 1% of the total number of spinal interneurons that project to each individual region) would be sufficient to generate the appropriate knee-hip synergy, even if they are most strongly activated during pocket scratching and synapse onto motoneurons. Thus, if indeed the pocket scratch knee-hip synergy is generated by interneurons with dual outputs to the knee and hip motoneurons, many such interneurons might indirectly affect one or both motor pools (e.g., disynaptically), *via* synapses onto intervening interneurons. Interneurons with such indirect projections would not have been double-labeled in the current study.

It is also possible that dual-projecting interneurons that are rhythmically active with hip-related timing contribute to generating the pocket scratch knee-hip synergy (either *via* direct or indirect projections) along with additional interneurons that are dedicated to knee-extensor control, as suggested by the mathematical exploration of the dual-projection circuit (Snyder and Rubin, 2015). Interneurons with specifically knee-related rhythmic activity were proposed as part of Grillner’s unit burst generator hypothesis (Grillner, 1981) and have been demonstrated during rostral scratching in the turtle spinal cord (Stein and Daniels-Mcqueen, 2003), though it is not known how common they are in the turtle spinal cord or whether the timing of their bursts is the same with respect to knee motor nerve bursts during pocket scratching.

### Synergy Generation by Spinal Interneurons in Other Limbed Vertebrates

Dual-projecting spinal interneurons could potentially play a role in previously described muscle synergy generation for a variety of limb movements in diverse vertebrates. In adult frogs, extracellular recording of intermediate zone interneurons in the rostral part of the hindlimb enlargement along with spike-triggered averaging of electromyograms has shown that there are spinal interneurons that mono- or disynaptically activate different motor pools, which could contribute to muscle synergy generation (Hart and Giszter, 2010). This location is comparable to the turtle D8-D9 intermediate zone (Schotland and Tresch, 1997), where we found several dual-projecting interneuron somata in the current study (Figure 4). In neonatal mice, “motor synergy encoder” premotor interneurons (with heterogeneous gene expression) in the deep dorsal horn throughout the hindlimb enlargement synaptically activate multiple hindlimb motor pools, in rostral and caudal hindlimb enlargement segments (lumbar 2 and 5), likely *via* dual axon projections (Levine et al., 2014). The premotor interneurons projecting to both motor pools make up about 0.3% of the premotor interneurons projecting to one pool, in the same range as the dual-projecting interneuron percentage we describe here. Taken together, across limbed vertebrate studies, a small percentage of premotor interneurons in the ipsilateral deep dorsal horn and the intermediate zone (and in the current study, also the dorsal part of the ventral horn bilaterally) of hindlimb enlargement segments appear to project to multiple motor pools. Hand muscle synergy-generating premotor interneurons have also been found in monkey forelimb enlargement spinal cord by spike-triggered averaging of electromyograms, though the cross-sectional gray matter regions of recordings were not reported (Takei et al., 2017). Further research may reveal dual-projecting interneuron subtypes that activate distinct combinations of motor pools (Levine et al., 2014).

## Data Availability Statement

The raw data supporting the conclusions of this article will be made available by the authors, without undue reservation, to any qualified researcher.

## Ethics Statement

The animal study was reviewed and approved by the Institutional Animal Care and Use Committee of the University of Oklahoma.

## Author Contributions

AB designed the experiments. KN and TS performed the experiments. TG performed the imaging for figures. KN, TS, and AB analyzed the data. KN and AB wrote the article.

## Conflict of Interest

The authors declare that the research was conducted in the absence of any commercial or financial relationships that could be construed as a potential conflict of interest.
